# Supplementary material for: Estimating total human deaths from rabies: A statistical framework leveraging reports of rabies transmission from deceased organ and tissue donors
Source: PLoS Negl Trop Dis. 2026 Sep 21;20(9):e0014731. doi: 10.1371/journal.pntd.0014731 (PMC13592719; doi:10.1371/journal.pntd.0014731)
Supplement: Supplementary file 6 [file pntd.0014731.s005.zip › README.docx]

**README for Supplementary Files for**

**Estimating total human deaths from rabies: a statistical framework leveraging reports of rabies transmission from deceased organ and tissue donors**

Younjung Kim^1,2^, Junjie Chen^1,2^, Alex Yan^1,2^, Tarek Alrefae^1,2^, Sumali Bajaj^1,2^, Christl A. Donnelly^1,2*^

1 Department of Statistics, University of Oxford, Oxford, United Kingdom

2 Pandemic Sciences Institute, University of Oxford, Oxford, United Kingdom

* [christl.donnelly@stats.ox.ac.uk](mailto:christl.donnelly@stats.ox.ac.uk)

**Directory Structure**

Supplementary files include Supplementary Data, Code, Parameter Space Exploration, and output files from the analyses assessing the impact of changes in the proportion of rabies deaths among deceased donors relative to decedents in the general population (the k model) and changes in the diagnostic sensitivity among deceased donors relative to decedents in the general population (the gamma model). Both analyses were performed with a country-specific diagnostic sensitivity (Method A) or a shared diagnostic sensitivity across countries with the same canine-rabies status (Method B).

Supplementary files have the following hierarchical directory structure.

- Code_A (for the k model)
- Code_B (for the gamma model)
- Data
- Figure_Collection (for parameter space exploration)
- Folder: Result_A (for the k model) or Result_B (for the gamma model) /
- gof_B_CRE.csv
- gof_B_CRF.csv
- s_tbl_B_CRE.csv
- s_tbl_B_CRF.csv
- Subfolders [Country_Name]/
  - prof_all_AplusB.csv
- prof_A.csv:
- prof_B.csv
- rho_tbl_A.csv / rho_tbl_B.csv
- und_tbl_A.csv / und_tbl_B.csv
- s_tbl_A.csv / s_tbl_B.csv
- gof_A.csv
- gof_B.csv
- Figure_S1, S2, S3_[Country_Name].pdf

**Explanations of Output Files in Result_A (for the k model) or Result_B (for the gamma model)**

**1. Global Estimates**

gof_B_CRE.csv: Global goodness of fit for canine-rabies-endemic countries

gof_B_CRF.csv: Global goodness of fit for canine-rabies-free countries

s_tbl_B_CRE.csv: Diagnostic sensitivity shared among canine-rabies-endemic countries

s_tbl_B_CRF.csv: Diagnostic sensitivity shared among canine-rabies-free countries

**1. The Main Consolidated File: prof_all_AplusB.csv**

Location: [Country_Name]/

It contains the yearly estimates for $\rho_{y}$ (proportion of rabies deaths), $U_{y}$ (undiagnosed deaths from rabies), and $T_{y}$ (total deaths from rabies) for both Methods A and B.

| Column Name | Description |
| --- | --- |
| Year | The observational year. |
| method_label | Label for the method used  (e.g. "A: country-specific s", "B: shared s"). |
| k_key / gamma_key | Formatted string of the scaling factor tested (e.g. "0.25", "1.00"). |
| rho_hat | The maximum likelihood estimate (MLE) of the proportion of rabies deaths among decedents in the general population ($\rho_{y}$). |
| rho_lo / rho_hi | The lower and upper bounds of the 95% profile likelihood confidence interval for $\rho_{y}$. |
| und_hat | The estimated number of undiagnosed rabies deaths. |
| und_lo / und_hi | The lower and upper bounds of the 95% confidence interval for undiagnosed deaths. |
| k_val / gamma | The numeric scaling factor applied in the respective model. |
| confirmed | The reported number of rabies deaths for that year. |
| total_hat | Total estimated rabies deaths (und_hat + confirmed). |
| total_lo / total_hi | Lower and upper bounds of the 95% confidence interval for total rabies deaths. |

**2. Sensitivity Estimates: s_tbl_A.csv and s_tbl_B.csv**

Location: [Country_Name]/

Contains the estimated baseline diagnostic sensitivities (s) for decedents in the general population.

| Column Name | Description |
| --- | --- |
| method_label | Identifies whether the estimate is country-specific (A) or shared (B). |
| k_key / gamma_key | The formatted string version of the scaling factor. |
| s_hat | The maximum likelihood estimate for diagnostic sensitivity (s). |
| s_lo / s_hi | The lower and upper bounds of the 95% profile likelihood confidence interval for s. |

**3. Granular Trajectory Tables: rho_tbl_A.csv / rho_tbl_B.csv / und_tbl_A.csv / und_tbl_B.csv**

Location: [Country_Name]/

These files contain the exact same data as prof_all_AplusB.csv, but are preserved separately for debugging and intermediate plotting steps.

**4. Goodness-of-Fit Metrics: gof_A.csv and gof_B.csv**

Location: [Country_Name]/

These files contain Likelihood Ratio (deviance) test results comparing the fitted models against a saturated Poisson model.

| Column Name | Description |
| --- | --- |
| k_val / gamma_val | The numeric scaling factor tested. |
| ll_sat | The log-likelihood of the saturated/idealized model. |
| ll_fit | The log-likelihood of the proposed fitted model. |
| G2 | The Deviance statistic |
| df | Degrees of freedom for the chi-square test. |
| p_value | The p-value derived from the deviance statistic. |
| method | Identifies the method evaluated ("A" or "B "). |
| country | The country evaluated. |
| group | (Method B only) Identifies if the country belongs to the canine-rabies-free or canine-rabies-endemic group. |

**Caption for “Figure_Collection.pdf”**

The figure shows the log-likelihood deviance for each country across the joint parameter space of diagnostic sensitivity ($s$) and the proportion of deaths from rabies among decedents in the general population ($\rho$). The colours correspond to the deviance thresholds defined by the 95.0, 97.5 and 99.0% confidence intervals.
